# Hydrogen Bond-Induced Activation of Photocatalytic and Piezophotocatalytic Properties in Calcium Nitrate Doped Electrospun PVDF Fibers

**DOI:** 10.3390/polym15153252

**Published:** 2023-07-30

**Authors:** F. F. Orudzhev, D. S. Sobola, Sh. M. Ramazanov, K. Častková, D. A. Selimov, A. A. Rabadanova, A. O. Shuaibov, R. R. Gulakhmedov, M. G. Abdurakhmanov, K. M. Giraev

**Affiliations:** 1 Smart Materials Laboratory, Dagestan State University, St. M. Gadjieva 43-a, 367015 Makhachkala, Russia; 2 Central European Institute of Technology BUT, Purkyňova 656/123, 61200 Brno, Czech Republic; 3 Department of Physics, Faculty of Electrical Engineering and Communication, Brno University of Technology, Technická 2848/8, 61600 Brno, Czech Republic; 4 Amirkhanov Institute of Physics, Dagestan Federal Research Center, Russian Academy of Sciences, 367003 Makhachkala, Russia

**Keywords:** PVDF, salt, hydrogen bond, exciton, photocatalysis, piezophocatalysis, visible light

## Abstract

In this study, polyvinylidene fluoride (PVDF) fibers doped with hydrated calcium nitrate were prepared using electrospinning. The samples were analyzed using scanning electron microscopy (SEM), X-ray diffraction (XRD), optical spectroscopy, X-ray photoelectron spectroscopy (XPS), Fourier transform infrared (FTIR), Raman, and photoluminescence (PL) spectroscopy. The results are complementary and confirm the presence of chemical hydrogen bonding between the polymer and the dopant. Additionally, there was a significant increase in the proportion of the electroactive polar beta phase from 72 to 86%. It was shown that hydrogen bonds acted as a transport pathway for electron capture by the conjugated salt, leading to more than a three-fold quenching of photoluminescence. Furthermore, the optical bandgap of the composite material narrowed to the range of visible light energies. For the first time, it the addition of the salt reduced the energy of the PVDF exciton by a factor of 17.3, initiating photocatalytic activity. The calcium nitrate-doped PVDF exhibited high photocatalytic activity in the degradation of methylene blue (MB) under both UV and visible light (89 and 44%, respectively). The reaction rate increased by a factor of 2.4 under UV and 3.3 under visible light during piezophotocatalysis. The catalysis experiments proved the efficiency of the membrane design and mechanisms of catalysis are suggested. This study offers insight into the nature of chemical bonds in piezopolymer composites and potential opportunities for their use.

## 1. Introduction

The pollution of wastewater is a serious problem that affects the health of humans, animals, and the environment. There are many good and highly effective methods of purification [1,2,3]; however, environmental requirements and the demand for renewable energy sources bring advanced oxidation processes (AOPs) to the forefront of science, among which photocatalysis, Fenton catalysis, piezocatalysis, pyrocatalysis, sonocatalysis, microbubble ozonation and others are of the greatest interest from both practical and fundamental points of view [4,5,6,7,8,9,10,11,12,13]. All these processes are based on the conversion of various types of natural green energy into useful chemical energy.

All heterogeneous processes occur at the interface and therefore the dimensionality of catalysts and their specific surface area play key roles in catalytic efficiency. In this regard, researchers’ efforts are directed towards obtaining low-dimensional ultradispersed catalysts [14,15,16]. However, the use of such catalysts creates practical difficulties associated with separating the catalysts from the purified water and the secondary pollution of water with nanoparticles [17,18,19]. Moreover, restoring the catalyst after separation may require expensive processing and incur significant time costs. To overcome this limitation, several approaches have been proposed, including magnetic separation of modified catalysts, immobilization of catalysts on substrates with a high specific surface area, and coating deposition [20,21,22].

Recently, the development of photocatalytic polymeric membranes has become a hot topic for overcoming the drawback of suspended photocatalysts, especially because of the possibility of its combining with membrane filtration and adsorption [21]. Photocatalysis using polymers, such as polyvinylidene fluoride (PVDF), has attracted considerable attention in recent years [23,24,25] for its non-toxicity, biocompatibility, ease of processing, and chemical, mechanical, and thermal stability [26]. However, given its inertness, one strategy for using PVDF is to synthesize heterophase composites by combination with functional semiconductor photocatalysts to impart functional properties [27,28,29,30,31]. Recently, the piezoelectricity of PVDF has been actively used in photocatalysis by creating a piezoelectric field in the membrane through mechanical action using ultrasound and water flow. This approach allows for the enhancement of the photocatalytic activity of PVDF/photocatalyst composites by spatially separating photo-generated electron-hole pairs [31,32,33]. However, long-term ultrasound treatment can lead to polymer degradation and the release of photocatalytic nanoparticles into the solution [21]. In this regard, finding ways for homogeneous functionalization of the PVDF polymer structure without adding functional nanoparticles is of interest. In our previous work, we demonstrated that the addition of CTAB leads to activated PVDF photocatalytic activity [34]. One interesting and accessible strategy in this direction is to create hydrogen bonds in the polymer matrix. Recent studies have reported that tuning hydrogen bonds in the structure of conjugated photocatalysts is one of the determining factors in enhancing photocatalytic activity by creating an effective pathway for transporting photo-generated electrons, leading to suppression of charge carrier recombination [35,36].

Earlier reports discussed the formation of hydrogen bonds in the structure of PVDF. In a study [37], detailed quantum chemical calculations revealed that improper C–H⋯O and proper O–H⋯F hydrogen bonds were formed between the hydrated salt molecule and the polymer chains: (PVDF/Al(NO_3_)_3_∙9H_2_O, PVDF–TrFE/Al(NO_3_)_3_∙9H_2_O, PVDF–HFP/Al(NO_3_)_3_∙9H_2_O), leading to changes in the highest occupied molecular orbital (HOMO)/lowest unoccupied molecular orbital (LUMO) energy levels. Another study [38] showed that incorporating Zn(NO_3_)_2_·6H_2_O, Mg(NO_3_)_2_·6H_2_O, MgCl_2_·6H_2_O, and AlCl_3_·6H_2_O into the PVDF matrix increased the electroactive phase by forming H-bonds between the filler and PVDF through electrostatic interactions. Experimental studies [30,39,40,41,42,43,44,45,46,47,48,49,50] also showed that the formation of hydrogen bonds (HB) between polymer chains and interstitial water molecules within the crystal of the hydrated salt caused structural changes in the polymer chains.

Considering all of the above and the broad prospects for the application of PVDF in ecology and biomedicine, and considering the fact that calcium plays a huge role in biological processes, PVDF nanofibers doped with hydrated calcium salt (PVDF/CN) were synthesized in this study. It was demonstrated for the first time that modification led to the initiation of photocatalytic activity in a PVDF polymer.

## 2. Materials and Methods

**Figure 1:**
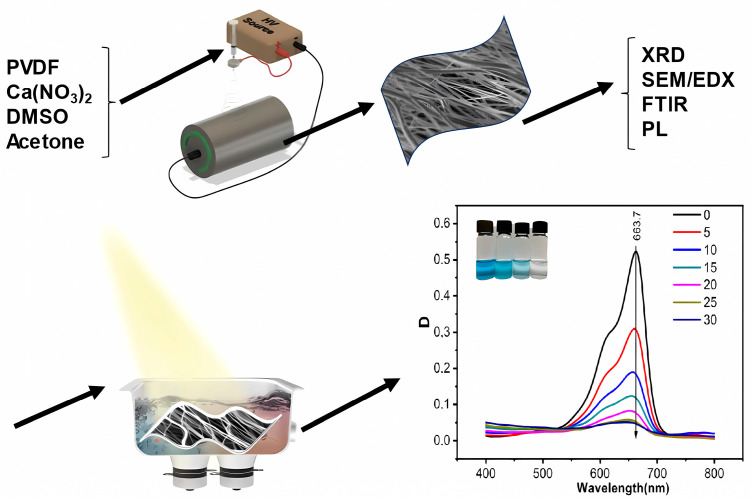
Schematic representation of the process of synthesis, analysis, and application.

A 15 wt.% solution of PVDF (Sigma Aldrich, St. Louis, MI, USA, Mw = 275,000 g·mol^−1^) was prepared in a mixture of dimethyl sulfoxide (Sigma Aldrich, St. Louis, MI, USA) and acetone (Sigma Aldrich, St. Louis, MI, USA) with a volumetric ratio of 7:3. Calcium nitrate (in hydrated form) (Lach-Ner, Neratovice, Czech Republic) was dissolved in the solvent for mixing at a concentration of 8 wt.% relative to the solid polymer prior to the dissolution of PVDF. The resulting solution was then subjected to electrospinning at a constant voltage of 50 kV using a 4-SPIN apparatus (Contipro, Dolní Dobrouč, Czech Republic) at a feed rate of 30 μL·min^−1^ with a needle diameter of 1.067 mm (17 G). The rotating collector was covered with aluminium foil to collect the resulting fibers at 2000 rpm for 30 min while maintaining a distance of 20 cm between the needle tip and the collector. The resulting nonwoven fiber mats were left to dry overnight at room temperature. Schematically, the procedure for synthesis, analysis and use is shown in Figure 1.

X-ray photoelectron spectroscopy (XPS) was performed to determine the types of chemical bonds in the samples using an AXIS Supra instrument (Kratos Analytical Ltd., Manchester, UK), with results recorded at an emission current of 15 mA and a resolution of 20 for wide spectra and 80 for element-specific spectra. The obtained spectra were fitted using CasaXPS software (version 2.3.23, Kratos Analytical Ltd., Manchester, UK) using Gaussian–Lorentzian line shapes.

FTIR data were obtained using a Bruker instrument (Billerica, MA, USA) in transmission mode with 512 scans at a resolution of 1 cm^−1^. X-ray diffraction (XRD) was conducted to confirm the crystalline structure of the samples using a Rigaku SmartLab 3 kW instrument (Rigaku, Tokyo, Japan) in Bragg–Brentano configuration with Cu Kα radiation.

Energy dispersive X-ray spectroscopy (EDX) was performed to track the homogeneity of element distribution and conducted at an accelerating voltage of 15 kV to provide an overall view of the element distribution on the surface of the fiber samples. The Tescan LYRA3 electron microscope (Tescan, Brno, Czech Republic) with an X-Max50 EDS detector from Oxford Instruments was used.

Measurements of total light transmission T_t_ and diffuse reflection Rd spectra for the investigated objects were conducted in the λ~300–1000 nm wavelength range using an Avasphere-50 integrating sphere (Avantes, Apeldoorn, The Netherlands). The light source was AvaLight-DH-S-BAL combined with a deuterium/halogen lamp (Avantes, Apeldoorn, Netherlands), and the radiation was delivered to the sample via an optical fiber having a diameter of 600 μm. Photocurrents were recorded using an automated MS3504i spectrometer (SOL-Instruments, Minsk, Belarus) in combination with an HS-101(HR)-2048 × 122 CCD array (Hamamatsu, Shizuoka, Japan) and a personal computer.

The final data for the spectrophotometric coefficients T_t_ and R_d_ were determined as follows (1):

(1) $$R_{d}^{exp}=\frac{R_{d}^{s}\left(\lambda \right)-R_{0}\left(\lambda \right)}{R_{gl}\left(\lambda \right)-R_{0}\left(\lambda \right)}\;\mathrm{and}\;T_{t}^{exp}=\frac{T_{t}^{s}\left(\lambda \right)-T_{0}\left(\lambda \right)}{T_{gl}\left(\lambda \right)-T_{0}\left(\lambda \right)},$$

where $T_{t}^{s}\left(\lambda \right)$ and $R_{d}^{s}\left(\lambda \right)$ are the transmission and reflection spectra of the samples; $T_{gl}\left(\lambda \right)$ and $R_{gl}\left(\lambda \right)$ are the spectra of the reference signal measured with quartz plates; $T_{0}\left(\lambda \right)$ is the signal of the integrating sphere with closed input and open output ports; and $R_{0}\left(\lambda \right)$ is the signal for the sphere with open optical ports. The spectral dependence of the optical absorption coefficient *μ_a_* and light scattering coefficient $\mu_{s}^{\prime }$ was calculated using an inverse Monte Carlo numerical modelling method, which is developed and described in detail in [51].

Microfluorescence properties were studied using a laser-scanning confocal microscope MP-350 (SOL-Instruments, Minsk, Belarus), as well as an optical microscope Nikon-NiU (Nikon, Tokyo, Japan), with excitation by laser light at a wavelength of λ exc = 408 nm and detection using a spectrometer MS3504i.

The piezophotocatalytic decomposition test was carried out using UV-visible and visible light radiation. A 250 W high-pressure mercury lamp (Philips, Amsterdam, The Netherlands) was used as the UV-visible light source, while a 70 W metal halide lamp (Osram, Munich, Germany) was used as the visible light source. The distance between the light source and the reactor was 10 cm. Piezo- and piezophotocatalytic decomposition were performed in an ultrasonic bath with a power of 250 W at a frequency of 18 kHz (Piezocatalysis, Piezophotocatalysis) and with stirring of the solution on a magnetic stirrer at a speed of 250 rpm (Stirring-Piezocatalysis, Stirring-Piezophotocatalysis). To eliminate the influence of temperature on the decomposition efficiency, a constant temperature of 26 °C was maintained in the reactor.

Before the test, a film (3 × 1 cm^2^) was immersed in a glass containing a solution of methylene blue (MB) (2.5 mg/L, 20 mL) and kept in the dark for 1 h to establish adsorption–desorption equilibrium. During the test, 3 mL of the solution was taken at certain time intervals and analysed using a UV-visible spectrometer. The concentration of MB dye was measured at its maximum absorption peak: λ = 663.7 nm. The percentage of decomposition was denoted as C/C_0_ (C and C_0_ are the measured and initial concentration of the dye solution, respectively). To distinguish the contributions from different factors affecting the decomposition, additional experiments were conducted on the decomposition of MB under ultrasound without catalyst (sonolysis), under ultrasound with catalyst (US-Piezocatalysis and Stirring-Piezocatalysis), and under light without catalyst (Photolysis-UV-Vis and Photolysis-Vis).

To understand the mechanism of piezophotocatalytic reactions under UV-visible radiation, experiments were carried out to capture the active particles generated in the system. Isopropanol (IPA), p-benzoquinone (p-BQ), ethylenediaminetetraacetic acid (EDTA), and silver nitrate (AgNO_3_) were used as scavengers of hydroxyl radicals (∙OH), superoxide radicals (∙O_2_^–^), holes (h^+^), and electrons (e^–^), respectively.

## 3. Results and Discussion

**Figure 2:**
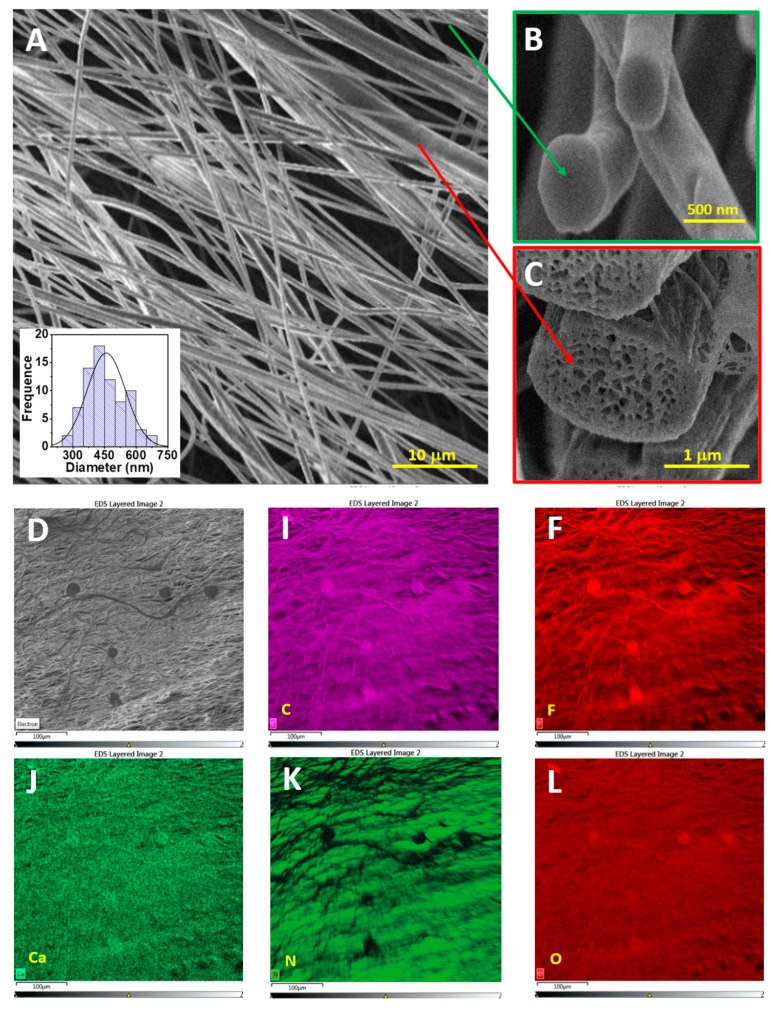
SEM of PVDF/CN fibers: (**A**) General view from above (**B**) Cross section of a small diameter individual fiber (**C**) Cross section of a large diameter individual fiber (**D**–**L**) EDS images of elemental mapping: C, F, Ca, N, O.

The morphology of the obtained materials was characterized using a scanning electron microscope (SEM), and the results are presented in Figure 2. It can be seen that the polymer consisted of elongated oriented nanofibers of varying diameters. The diameter of the spun fibers ranges from 300 to 700 nm (Figure 2B). High-resolution SEM images (Figure 2A, green and red areas on the right) revealed that fibers with a diameter of up to 500 nm are solid and homogeneous, while larger diameter fibers are porous and have a more developed and rougher surface. The histogram shows the distribution of fibers by diameter. Elemental mapping of Figure 2D shows a homogeneous distribution of C, F, Ca, O, N elements and the absence of contaminants. 

It is known from the literature that PVDF has five crystalline phases, among which the most well-known and studied are the α, β, and γ [52]. In the α-phase, the macromolecules are arranged in a TGTG sequence that forms a loosely twisted spiral with CF_2_ dipoles pointing in different directions, resulting in a close-to-zero net polarization in the monoclinic crystal cell. The PVDF macromolecules in the β-phase form a flat zigzag pattern, with a TT′TT′ sequence and CF_2_ dipoles in neighboring links pointing in the same direction. The elementary unit cell of the β-phase is orthorhombic and has a constant dipole moment. The γ-form of PVDF also has an orthorhombic elementary unit cell. In the γ-phase, the links have a T_3_GT_3_G′ sequence with an uncompensated dipole moment although smaller than in the β-phase. An X-ray diffraction analysis was conducted to determine the crystalline structure.

**Figure 3:**
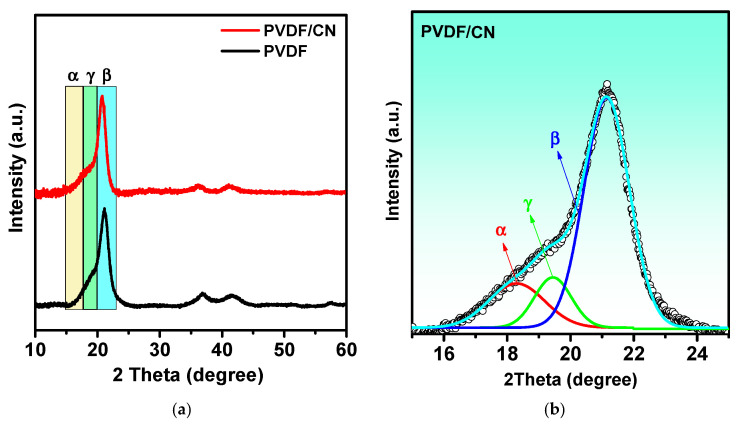
(**a**) XRD spectra of PVDF and PVDF/CN; (**b**) Deconvolution of PVDF/CN peak.

**Figure 4:**
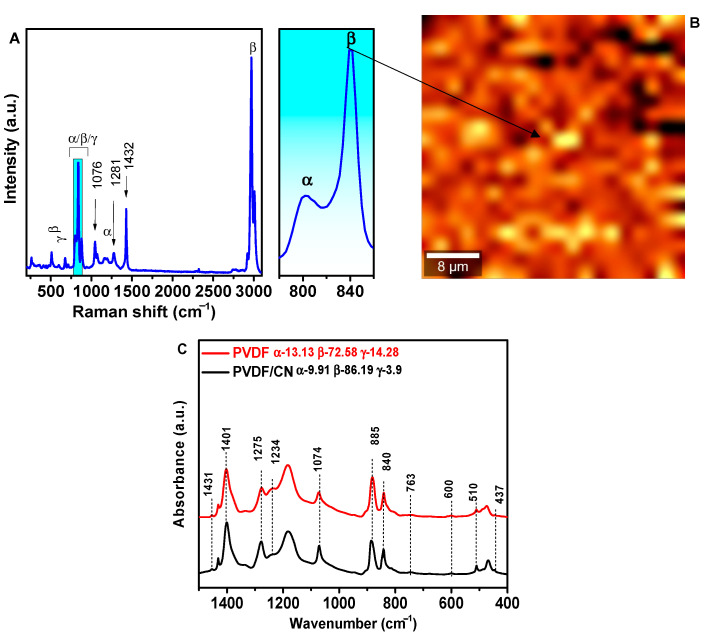
(**A**) Raman spectrum of PVDF/CN. (**B**) Raman mapping of the region around the 840 cm^−1^ peak. (**C**) IR-Fourier spectra of PVDF/CN in the range of 400–1500 cm^−1^.

Figure 3a shows the XRD spectra for PVDF and PVDF/CN, where distinct peaks are observed at diffraction angles of 21.2° and 59.2° corresponding to the β-polymorphic phase [53]. The peak at 36.6° is attributed to both β- and γ-phases. Two peaks at angles of 20.9° and 41.8° are related to the α-phase [54]. Figure 3b shows the Gaussian decomposition of the shoulder at an angle of 18.9°, indicating that the three phases are located very close to each other [55], making quantitative phase analysis difficult. To perform quantitative analysis of the phase states, it is necessary to use Raman spectroscopy and IR-Fourier analysis. Figure 4A shows the Raman spectrum of a PVDF/CN composite. The structural difference between α- and β-phases is usually associated with the Raman lines related to CH_2_ group wagging vibrations at 795 cm^−1^ for α-phase and 840 cm^−1^ for β-phase, accompanied by CF_2_ skeletal bending vibrations at 610 cm^−1^ and 510 cm^−1^ for α- and β-crystalline phases, respectively [56]. The peak at 1076 cm^−1^ was attributed to CC asymmetric stretching; peaks at 1281 cm^−1^ were characteristic of CC symmetric stretching and CCC skeletal bending; while the peak at 1432 cm^−1^ corresponded to CH_2_ skeletal bending and CH_2_ rocking [57]. The most characteristic region in Raman spectroscopy for evaluating phase distribution is the region of 780–860 cm^−1^, presented in the inset of Figure 4A. It can be seen that the β-phase is predominant as manifested by a characteristic peak at 840 cm^−1^. Figure 4B shows a Raman mapping image of the region around the 840 cm^−1^ peak, where bright areas correspond to peak intensity.

The relative content of all three α-, β-, and γ-phases in the samples was calculated from the IR-Fourier spectra. The results are presented in Figure 4C. The fraction of the electroactive phase was calculated according to Equation (1), where I_EA_ and I_763_ are the absorption at 840* and 763 cm^−1^, respectively; K_840*_ and K_763_ are the absorption coefficients at the corresponding wave numbers, the values of which were 7.7 × 10^4^ and 6.1 × 10^4^ cm^2^ mol^−1^, respectively. Then, using Equation (2), the fraction of β- and γ-phases in the electroactive phase was calculated: ΔHβ′ and ΔHγ′ are the height differences (absorption differences) between peaks around 1275 cm^−1^ and the nearest valley around 1260 cm^−1^, and the peak around 1234 cm^−1^ and the nearest valley around 1225 cm^−1^, respectively.

The calculations showed that in pure PVDF, the fraction of α-phase was 13.13%, while β- and γ-phases were 72.58 and 14.28%, respectively. For doped samples, the fraction of α- and γ-phases decreased to 9.91 and 3.9%, respectively, while the content of β-phase increased to 86.19%. The calculation shows that transconformational transitions mainly occurred from γ-T3GT3G′ (trans-trans-trans-gauche-trans-trans-trans-gauche′) to the β-TGTG′ (trans-gauche-trans-gauche′) polymorph, which is associated with the formation of hydrogen bonds. Due to the presence of water molecules in the structure of the hydrated calcium alt in PVDF/CN, hydrogen bonds can be formed between CF_2_ groups and H_2_O –C-F…H–O–H. In [58], the formation of hydrogen bonds in the PVDF structure upon the addition of hydrated magnesium salt was experimentally confirmed using IR spectroscopy. Theoretical quantum chemical calculations in [37] for PVDF/Al(NO_3_)_3_∙9H_2_O, PVDF–TrFE/Al(NO_3_)_3_∙9H_2_O, and PVDF–HFP/Al(NO_3_)_3_∙9H_2_O complexes also confirm the existence of hydrogen bonds between the salt molecule and polymer chains.

**Figure 5:**
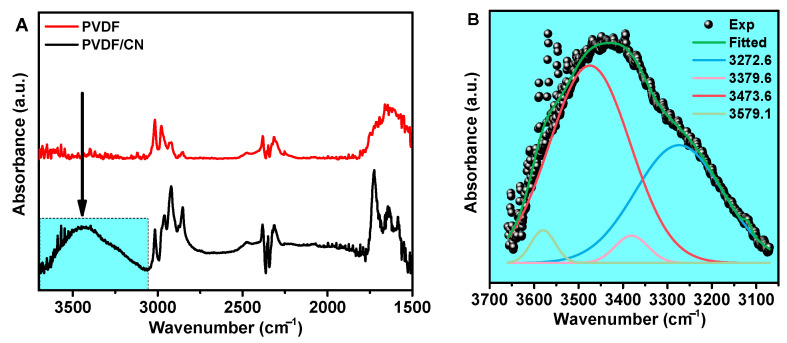
(**A**). IR-Fourier spectra in the range of 1500–3750 cm^−1^ for PVDF and PVDF/salt. (**B**). IR-Fourier spectra in the range of 3100–3750 cm^–1.^

To prove the hypothesis of hydrogen bond formation, FTIR spectra of pure PVDF and PVDF/CN in the range of 1500–4000 cm^−1^ were compared as shown in Figure 5A. The region of particular interest in this regard is 3100–3600 cm^−1^, which characterizes OH stretching modes [59,60].

For pure PVDF fibers without a salt additive, no peaks were observed in the 3100–3600 cm^−1^ range, while for PVDF with salt, a broad asymmetric peak centered at 3450 cm^−1^ was evident. In [61], it was shown that the analysis of IR spectra in the range of 3100–3600 cm^−1^ can be used to characterize hydrogen bonding interactions in aqueous solutions. It was demonstrated that the broad peak can be adequately described by four Gaussian bands of OH valence vibrations. Two peaks at 3272.6 and 3379.6 cm^−1^ can be attributed to water molecules that are fully hydrogen bonded. Two additional deconvoluted bands observed at approximately 3530 and 3605 cm^−1^ in the infrared spectrum corresponded to vibrations of partially hydrogen-bonded water molecules (Figure 5B). In [58], it was also suggested that doping PVDF with magnesium nitrate of different masses would result in a peak at 3261 cm^−1^ corresponding to water hydrogen-bonded to the polymer matrix and a peak at 3401 cm^−1^ corresponding to free water.

XPS measurements were done to obtain information on the material’s chemical composition after doping.

**Figure 6:**
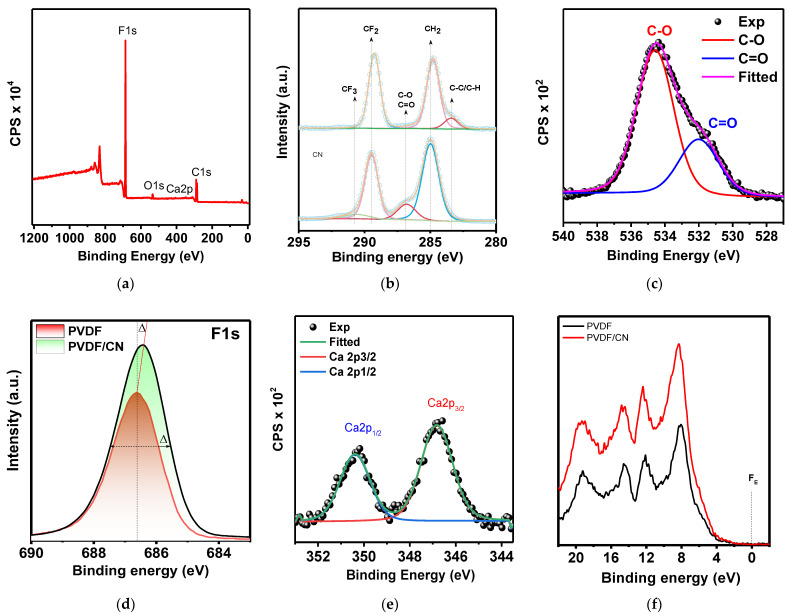
XPS spectra of the sample: (**a**) survey XPS spectrum, (**b**) detailed C1s peak, (**c**) detailed O1s peak, (**d**) detailed F1s peak, (**e**) detailed Ca2p peak, (**f**) valence band spectrum.

In Figure 6a, an overview spectrum of the PVDF/CN nanocomposite is presented, and peaks of the elements F, O, C, and Ca are detected. The C1s electron spectrum of pure PVDF consisted of three Gaussian peaks with centers at 289.2, 284.8, and 283.4 eV, identified, respectively, as CF_2_, CH_2_, and C–C/C–H (Figure 6b). For PVDF, the ratio of integral areas CH_2_:CF_2_ was 1.59, while for PVDF/CN it is 0.86. Moreover, the peaks shift towards lower energies after doping. All this together indicated the chemical bonding of CH_2_:CF_2_ functional groups of the polymer with calcium nitrate. However, the peak at 286 eV corresponding to C–O/C=O, which is absent in pure PVDF, appears for PVDF/CN, while the peak at 283.4 corresponding to C–C/C–H, characteristic of pure PVDF, practically disappears for PVDF/CN. The O1s spectrum for PVDF/CN presented in Figure 6c consisted of two peaks at 534.57 and 532.0 eV, characterizing two types of oxygen bonds C–O and C=O, confirming the results of C1s [62]. The Ca2p element spectrum in Figure 6e shows that calcium is in the oxidation state of two Ca^2+^ and serves as a check for the presence of salt in the sample, along with EDX elemental mapping. The F1s level spectra presented in Figure 6d were characterized by one peak at 686.6 eV and 686.3 eV for pure and doped PVDF, respectively [34]. The presence of the hydrogen bond C–F....H–O led to the redistribution of electronic density, resulting in the weakening of the binding energy of 1s electrons in CF_2_ on the F1s spectrum. As a result, we observed a characteristic shift towards lower energies. Similar observations were made in other studies [63]. The valence band (VB) XPS spectrum in Figure 6f shows that the position of the valence band after doping changed from 4 to 3.7 eV. Thus, the XPS results indirectly confirmed the conclusions about the formation of hydrogen bonds in the PVDF/CN composite [64].

**Figure 7:**
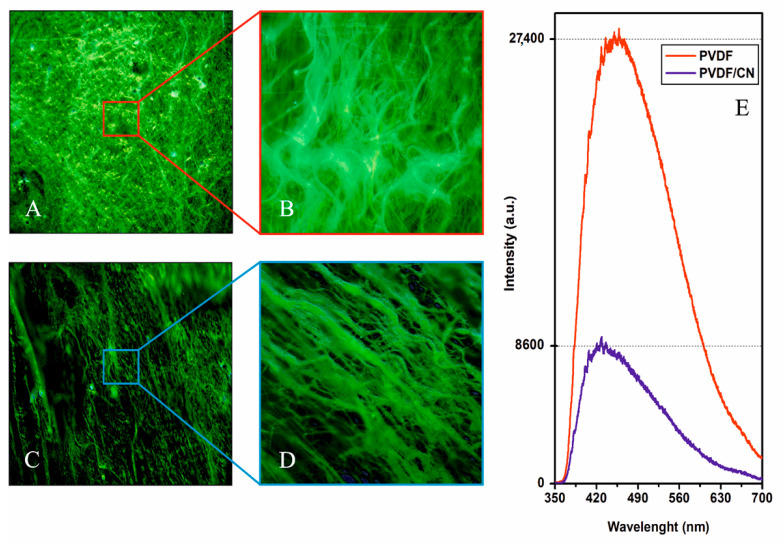
Fluorescence micrographs of (**A**,**B**) PVDF (**C**,**D**) PVDF/CN at 500× magnification, (**E**) photoluminescence spectra.

For materials used as photocatalysts, one of the important characteristics that determines the potential applicability of the material is its optical properties. Photoluminescence is a non-destructive method used mainly to detect the efficiency of capturing photo-generated charge carriers. The results of the micro fluorescence studies of samples are shown in Figure 7. As can be seen from Figure 7A,B, when excited at a wavelength of 408 nm, the objects were characterized predominantly by yellow–green fluorescence. Morphological structure elements in the form of fibers are clearly visible.

**Figure 8:**
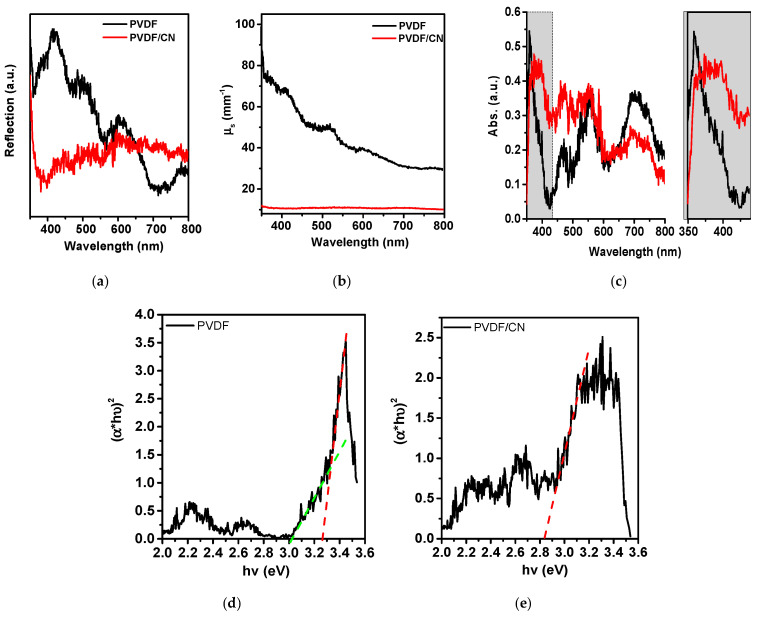
Optical spectra of the samples (**a**) reflectance, (**b**) scattering, (**c**) absorbance, (**d**,**e**) Tauc curves.

As a result of exciting the polymer matrix with ultraviolet radiation, a photoluminescence spectrum was observed in a wide range of radiation from 360 to 700 nm (Figure 7C) with a maximum in the green region and a shoulder in the yellow. It can be seen that pure PVDF demonstrated a strong PL signal, which characterized a radiative recombination of photo-generated charges formed due to electronic transition from its conduction band to the valence band. However, the intensity of the PL of the PVDF/CN sample was significantly reduced due to a low rate of charge recombination and high efficiency of separation. Spectral characteristics of optical coefficients of reflection, transport scattering, and absorption are presented in Figure 8. 

The reflection spectrum in Figure 8a shows that pure PVDF had a strong reflectivity in the range with a maximum at 420 nm and decreasing to 700 nm. Doped PVDF exhibited a dip in the 400 nm range with an increasing reflection coefficient up to 800 nm. Additionally, Figure 8b shows that pure PVDF had high transport-scattering coefficient values. Up to 420 nm, the transport-scattering coefficient of pure PVDF was 7–8 times higher than that of the doped PVDF. The calculated spectra of the absorption coefficient are presented in Figure 8c where doping resulted in a red shift. Using the Tauc curve in the coordinates for direct interband transition, the values of optical bandgap were calculated, which are 3.27 and 2.84 eV for pure and doped PVDF, respectively.

The above results indicated that PVDF/CN can be used as a catalyst in photo- and piezophotocatalytic processes. The material was tested as a catalyst in MB decomposition under UV-visible and visible light irradiation. The data obtained under UV-visible irradiation are presented in Figure 8a,b.

**Figure 9:**
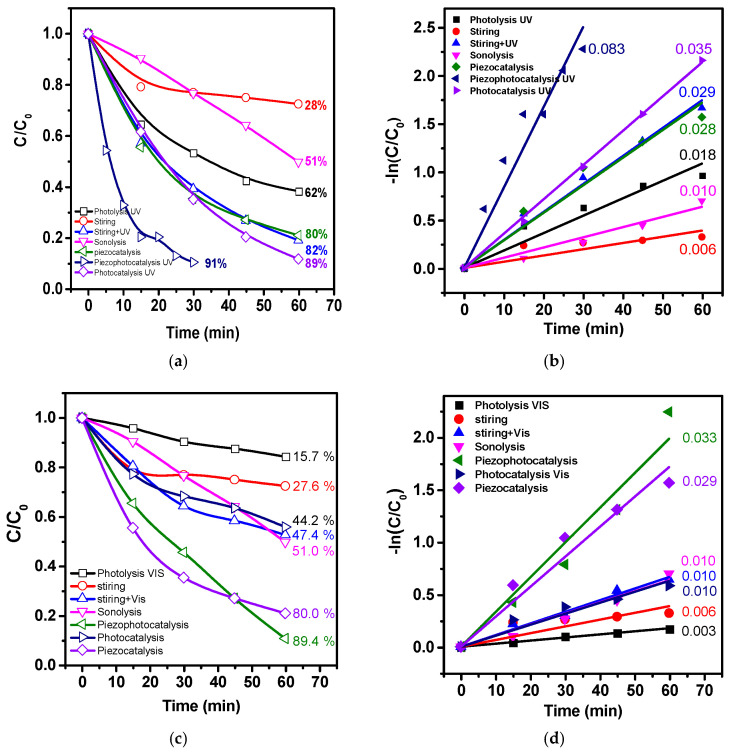
Catalytic activity of PVDF/CN under UV-Vis (**a**) and visible light (**c**) and pseudo-first ordering kinetic curves (**b**,**d**) respectively.

Figure 9 depicts the results of several experiments conducted on the degradation of dye molecules in a composite material consisting of PVDF and a catalyst. First, blank experiments were carried out using sonolysis (ultrasound treatment without catalyst) and photolysis (light treatment without catalyst). As shown in Figure 9a, after 60 min of sonolysis and photolysis, 51 and 62% of the dye had degraded, respectively. Next, experiments were conducted using photocatalysis with the composite material. The experiment was performed without any stirring to exclude the effect of possible piezopotential generation in the film. The results showed that the composite material exhibited high photocatalytic activity, with 89% of the dye degrading after 60 min. Under similar conditions, the degree of degradation for pure PVDF was only 24%, indicating that PVDF did not exhibit photoactivity. The reduced degradation compared to photolysis was likely due to the reflective and light-scattering properties of the pure polymer and strong radiative recombination. 

The piezocatalytic activity in the dark was investigated under mechanical stress induced by ultrasound or water flow during stirring of the solution by a magnetic stirrer. The degree of dye degradation under these conditions was 80 and 28%, respectively. The increased activity compared to blank experiments suggested that a piezopotential was generated in the composite, leading to a cascade of redox reactions that resulted in the degradation of dye molecules. Piezophotocatalysis showed the highest activity, with 91% degradation in 30 min. Rate constants (k) were calculated from the kinetic curves shown in Figure 8b using the Langmuir–Hinshelwood pseudo-first-order equation. The values of k were 0.018, 0.010, 0.028, 0.083, and 0.035 min^−1^ for photolysis, sonolysis, piezocatalysis, piezophotocatalysis, and photocatalysis, respectively. The reaction rate increased under piezophotocatalysis (simultaneous treatment with ultrasound and UV-visible light) by 4.6, 8.3, 3, and 2.3 times compared to photolysis, sonolysis, piezocatalysis, and photocatalysis, respectively, which indicated a piezophototronic effect.

**Figure 10:**
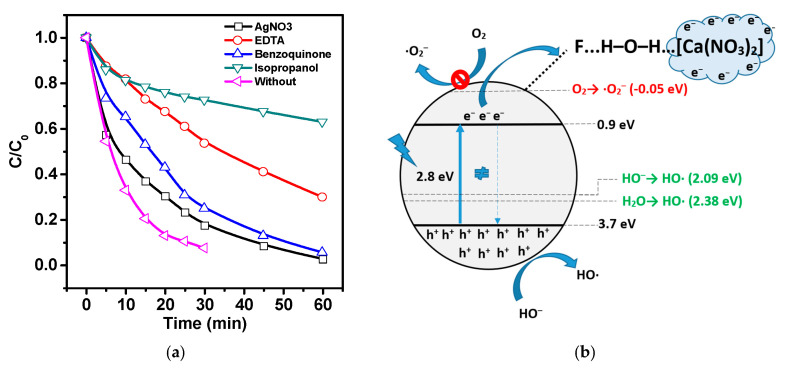
(**a**). Piezophotocatalytic activity of PVDF/CN under UV-visible light with scavengers, (**b**). Schematically represented energy band level diagram and degradation mechanism.

Taking into account the bandgap energy, similar experiments were conducted under visible light (see Figure 9c,d). The degree of decomposition during photolysis was about 16% in 60 min, indicating a high stability of the dye to visible radiation. It could be concluded that photolysis did not play a major role during heterogeneous photocatalytic experiments. The degree of decomposition of about 44% in the experiment indicated good photocatalytic activity of the composite under visible light. The degree of decomposition slightly increased in piezophotocatalysis compared to piezocatalysis (80 and 89%), indicating that the piezophototronic effect was not activated under visible light. To determine the reaction mechanism, experiments were conducted for piezophotocatalysis under UV-visible light to capture active redox forms. The results are presented in Figure 10.

When discussing the mechanism of using light energy in polymer photocatalysts, it is important to consider that low values of dielectric permeability and significantly higher exciton (e^–^–h^+^)^0^ binding energies impose certain limitations on the applicability of the classical model of photocatalysis mechanism based on free charge carriers [65]. In this regard, it is important to assess the exciton energy in PVDF and understand how it is influenced by the addition of calcium nitrate. Since photoluminescence can essentially be considered as an exciton recombination during which the excited electron returns to the valence band, the energy of the exciton can be roughly estimated based on the difference between the energy of the optical band gap and the energy of photoluminescence emission. Based on the optical data presented above, the exciton energy for pure PVDF and PVDF/CN was estimated as 520 and 30 MeV, respectively. As can be seen, the PVDF charges experience Coulomb attraction exceeding thermal energy k_B_T = 25 meV by an order of magnitude, indicating that excitons were the main product of charge excitation. For PVDF/CN, the exciton energy was comparable to thermal excitation energy, indicating that free charge carriers were the main product of charge excitation. The addition of calcium nitrate, which is hydrogen-bonded to the PVDF molecular chain, led to exciton dissociation. It was evident that calcium nitrate acted as an effective electron acceptor, while hydrogen bonds provided a transport pathway for electron transfer. This was also clearly demonstrated by the quenching of photoluminescence (see Figure 7E). The mechanism of exciton dissociation into free charge carriers in the presence of excited, delocalized zone states near the donor–acceptor interface in organic photovoltaic devices had been reported in previous studies [66,67]. Thus, the classical model, based on free charge carriers, can be correctly applied to explain the photocatalysis mechanism in PVDF/CN. To determine the most probable reaction pathway, it was necessary to determine the presence of electrons, holes, and active radicals: ·O_2_^−^ and ·OH in solution during the experiment. It should be understood that radicals were generated in solution when e^–^ and h^+^ interacted with dissolved oxygen and water. In the case of combined piezophotocatalysis, electrons and holes in the catalyst were generated either directly from light irradiation with energy exceeding the bandgap energy or through ultrasound, in which case there was no consensus on the mechanism of electron–hole pair generation since it is not as obvious as in photocatalysis. However, the most likely mechanisms are

1. Indirect sonolysis through cavitation pyrolysis of water, leading to the generation of short-lived hydroxyl radicals upon bubble formation and collapse;
2. Photocatalytic mechanism (PCM), in which sonoluminescence during cavitation may contribute to the formation of electron–hole pairs;
3. Thermocatalytic mechanism (TCM), in which, according to the hot spot theory, local high temperature during cavitation may promote the formation of electron–hole pairs through thermo-induced thermal excitation of the semiconductor.

From Figure 9a, it can be seen that adding a hole and hydroxyl radical scavenger led to the strong inhibition of catalytic activity. This indicated that ·OH radicals, which were formed involving holes, played a key role in the decomposition of the dye. In addition, the superoxide anion radicals and electrons played a minor role in MB decomposition. Since the oxidizing-reducing ability of photo-generated charge carriers strongly depended on the arrangement of energy levels relative to the oxidizing-reducing potentials of active forms, the energy level arrangement was calculated using experimental data on VB XPS and the optical bandgap width. Figure 10b shows a possible reaction mechanism. It can be seen that CB is located below the potential of O_2_/·O_2_^−^, which makes the energy-inefficient process of restoring dissolved oxygen in the solution unfavorable. However, as shown in Figure 10a, adding p-benzoquinone slightly reduces catalytic activity. This effect can be explained by the fact that in the absence of selective reagents in the form of superoxide radicals, quinones may undergo partial photoreduction with the formation of ·OH radicals and semiquinone, as reported in [68]. A similar effect is achieved by adding silver nitrate a trap for electrons. The minor effect can be explained by the possible partial photoreduction of silver, as it is known that silver enhances photocatalytic properties. In addition, the position of the CB level makes the generation of ·OH radicals from water energetically favourable, which was also confirmed by the results with absorbers.

Summarizing the above, we can describe the following possible reaction mechanism:

PVDF/CN + hv → (e^–^–h^+^)^0^ → e^–^ + h^+^ (2)

PVDF/CN + US → e^–^ + h^+^ (3)

h^+^ + H_2_O → ∙OH + H^+^ (4)

∙OH + Methylene Blue → Reaction products(5)

Thus, it can be assumed that excited electrons are captured by calcium nitrate, which is hydrogen-bonded to fluorine in the PVDF structure. Considering the large difference in dipole moments between fluorine and hydrogen atoms, the bond will be highly polar, generating an internal electric field at the PVDF/CN interface that will act as a driving force for transporting generated charges through the hydrogen bond to the electron acceptor, thereby limiting recombination. Another mechanism limiting recombination was the generated internal piezoelectric field in the polymer. Recent studies have experimentally shown that the presence of hydrogen bonds in the structure of conjugated photocatalysts is one of the determining factors for enhancing photocatalytic activity because it creates an effective path for transporting photo-generated electrons, thereby leading to suppression of recombination.

## 4. Conclusions

Polyvinylidene fluoride (PVDF) fibers doped with hydrated calcium nitrate were prepared by electrospinning. The described composite fibers proved to be beneficial for water treatment, as demonstrated by the example of MB decomposition. This behavior was attributed to an increase in the electroactive phase compared to pure material (from 72 to 86%). The presence of dielectric domains created an internal electric field, which helped reduce charge recombination. The low recombination rate was confirmed by photoluminescence measurements. Additionally, the presence of hydrogen bonds enhanced the transport of photo-generated electrons. It was shown that hydrogen bonds acted as a pathway for electron capture by the conjugated salt, resulting in a more than three-fold decrease in photoluminescence. The existence of hydrogen bonds was confirmed through analysis of both FTIR and XPS data. It was the first time that the addition of salt had been shown to decrease the energy of PVDF excitons by 17.3 times, leading to the initiation of photocatalytic activity. It was also important to note that the doped sample exhibited a reduced band gap, making it suitable for catalysis under visible light. Therefore, this work demonstrated for the first time that tuning the polymer’s conjugation with an inorganic hydrated salt through hydrogen bonds can initiate high piezo-, photo-, and piezophotocatalytic activity in the polymer, which opens up prospects for the development of inexpensive and accessible hybrid catalysts. 

## Data Availability

Data can be provided upon request to the corresponding author.

## References

[1] Tran N.N., Escribà-Gelonch M., Sarafraz M.M., Pho Q.H., Sagadevan S., Hessel V. (2023). Process Technology and Sustainability Assessment of Wastewater Treatment. Ind. Eng. Chem. Res..

[2] De Andrade J.R., Oliveira M.F., Da Silva M.G.C., Vieira M.G.A. (2018). Adsorption of Pharmaceuticals from Water and Wastewater Using Nonconventional Low-Cost Materials: A Review. Ind. Eng. Chem. Res..

[3] Alkhadra M.A., Su X., Suss M.E., Tian H., Guyes E.N., Shocron A.N., Conforti K.M., De Souza J.P., Kim N., Tedesco M. (2022). Electrochemical Methods for Water Purification, Ion Separations, and Energy Conversion. Chem. Rev..

[4] Kokkinos P., Venieri D., Mantzavinos D. (2021). Advanced Oxidation Processes for Water and Wastewater Viral Disinfection. A Systematic Review. Food Environ. Virol..

[5] Ma D., Yi H., Lai C., Liu X., Huo X., An Z., Li L., Fu Y., Li B., Zhang M. (2021). Critical Review of Advanced Oxidation Processes in Organic Wastewater Treatment. Chemosphere.

[6] Liu P., Wu Z., Abramova A.V., Cravotto G. (2021). Sonochemical Processes for the Degradation of Antibiotics in Aqueous Solutions: A Review. Ultrason. Sonochem..

[7] Kumar A., Rana A., Sharma G., Naushad M., Dhiman P., Kumari A., Stadler F.J. (2019). Recent Advances in Nano-Fenton Catalytic Degradation of Emerging Pharmaceutical Contaminants. J. Mol. Liq..

[8] Chauhan R., Dinesh G.K., Alawa B., Chakma S. (2021). A Critical Analysis of Sono-Hybrid Advanced Oxidation Process of Ferrioxalate System for Degradation of Recalcitrant Pollutants. Chemosphere.

[9] Babuponnusami A., Muthukumar K. (2014). A Review on Fenton and Improvements to the Fenton Process for Wastewater Treatment. J. Environ. Chem. Eng..

[10] Wang N., Zheng T., Zhang G., Wang P. (2016). A Review on Fenton-like Processes for Organic Wastewater Treatment. J. Environ. Chem. Eng..

[11] Ramos M.D.N., Santana C.S., Velloso C.C.V., da Silva A.H.M., Magalhães F., Aguiar A. (2021). A Review on the Treatment of Textile Industry Effluents through Fenton Processes. Process Saf. Environ. Prot..

[12] Verinda S.B., Muniroh M., Yulianto E., Maharani N., Gunawan G., Amalia N.F., Hobley J., Usman A., Nur M. (2022). Degradation of Ciprofloxacin in Aqueous Solution Using Ozone Microbubbles: Spectroscopic, Kinetics, and Antibacterial Analysis. Heliyon.

[13] Moradi M., Elahinia A., Vasseghian Y., Dragoi E.N., Omidi F., Mousavi Khaneghah A. (2020). A Review on Pollutants Removal by Sono-Photo -Fenton Processes. J. Environ. Chem. Eng..

[14] Lv S., Du Y., Wu F., Cai Y., Zhou T. (2022). Review on LSPR Assisted Photocatalysis: Effects of Physical Fields and Opportunities in Multifield Decoupling. Nanoscale Adv..

[15] Retamoso C., Escalona N., González M., Barrientos L., Allende-González P., Stancovich S., Serpell R., Fierro J.L.G., Lopez M. (2019). Effect of Particle Size on the Photocatalytic Activity of Modified Rutile Sand (TiO_2_) for the Discoloration of Methylene Blue in Water. J. Photochem. Photobiol. A Chem..

[16] Wang H., Lu J. (2020). A Review on Particle Size Effect in Metal-Catalyzed Heterogeneous Reactions. Chin. J. Chem..

[17] Ahmad I., Zou Y., Yan J., Liu Y., Shukrullah S., Naz M.Y., Hussain H., Khan W.Q., Khalid N.R. (2023). Semiconductor Photocatalysts: A Critical Review Highlighting the Various Strategies to Boost the Photocatalytic Performances for Diverse Applications. Adv. Colloid Interface Sci..

[18] Joseph A., Vijayanandan A. (2023). Review on Support Materials Used for Immobilization of Nano-Photocatalysts for Water Treatment Applications. Inorganica Chim. Acta.

[19] Li X., Chen Y., Tao Y., Shen L., Xu Z., Bian Z., Li H. (2022). Challenges of Photocatalysis and Their Coping Strategies. Chem. Catal..

[20] Jacinto M.J., Ferreira L.F., Silva V.C. (2020). Magnetic Materials for Photocatalytic Applications—A Review. J. Solgel. Sci. Technol..

[21] Zakria H.S., Othman M.H.D., Kamaludin R., Sheikh Abdul Kadir S.H., Kurniawan T.A., Jilani A. (2021). Immobilization Techniques of a Photocatalyst into and onto a Polymer Membrane for Photocatalytic Activity. RSC Adv..

[22] Orudzhev F.F., Alikhanov N.M.-R., Rabadanov M.K., Ramazanov S.M., Isaev A.B., Gadzhimagomedov S.K., Aliyev A.S., Abdullaev V.R. (2018). Synthesis and study of the properties of magnetically separable nanophotocatalyst BiFeO_3_. Chemical Probl..

[23] Ghosh S., Kouamé N.A., Ramos L., Remita S., Dazzi A., Deniset-Besseau A., Beaunier P., Goubard F., Aubert P.H., Remita H. (2015). Conducting Polymer Nanostructures for Photocatalysis under Visible Light. Nat. Mater..

[24] Banerjee T., Podjaski F., Kröger J., Biswal B.P., Lotsch B.V. (2020). Polymer Photocatalysts for Solar-to-Chemical Energy Conversion. Nat. Rev. Mater..

[25] Dai C., Liu B. (2020). Conjugated Polymers for Visible-Light-Driven Photocatalysis. Energy Environ. Sci..

[26] Dallaev R., Pisarenko T., Sobola D., Orudzhev F., Ramazanov S., Trčka T. (2022). Brief Review of PVDF Properties and Applications Potential. Polymers.

[27] Dong C., Fu Y., Zang W., He H., Xing L., Xue X. (2017). Self-Powering/Self-Cleaning Electronic-Skin Basing on PVDF/TiO_2_ Nanofibers for Actively Detecting Body Motion and Degrading Organic Pollutants. Appl. Surf. Sci..

[28] Cauda V., Stassi S., Bejtka K., Canavese G. (2013). Nanoconfinement: An Effective Way to Enhance PVDF Piezoelectric Properties. ACS Appl. Mater. Interfaces.

[29] Hafiza N., Norharyati W., Salleh W., Rosman N., Asikin N. (2019). PVDF/Fe_2_O_3_ Mixed Matrix Membrane for Oily Wastewater Treatment. Malays. J. Fundam. Appl. Sci..

[30] Prasad G., Sathiyanathan P., Prabu A.A., Kim K.J. (2017). Piezoelectric Characteristics of Electrospun PVDF as a Function of Phase-Separation Temperature and Metal Salt Content. Macromol. Res..

[31] Wu W., Yin X., Dai B., Kou J., Ni Y., Lu C. (2020). Water Flow Drived Piezo-Photocatalytic Flexible Films: Bi-Piezoelectric Integration of ZnO Nanorods and PVDF. Appl. Surf. Sci..

[32] Cui Y., Yang L., Zheng J., Wang Z., Li B., Yan Y., Meng M. (2021). Synergistic Interaction of Z-Scheme 2D/3D g-C_3_N_4_/BiOI Heterojunction and Porous PVDF Membrane for Greatly Improving the Photodegradation Efficiency of Tetracycline. J. Colloid Interface Sci..

[33] Dai B., Huang H., Wang W., Chen Y., Lu C., Kou J., Wang L., Wang F., Xu Z. (2017). Greatly Enhanced Photocatalytic Activity by Organic Flexible Piezoelectric PVDF Induced Spatial Electric Field. Catal. Sci. Technol..

[34] Rabadanova A., Abdurakhmanov M., Gulakhmedov R., Shuaibov A., Selimov D., Sobola D., Částková K., Ramazanov S., Orudzhev F. (2022). Piezo-, Photo- and Piezophotocatalytic Activity of Electrospun Fibrous PVDF/CTAB Membrane. Chim. Techno Acta.

[35] Jiang R., Lu G., Dang T., Wang M., Liu J., Yan Z., Xie H. (2023). Hydrogen-Bond Based Charge Bridge in a Heterojunction System for the Synergistic Degradation and Detoxification of Two PPCPs. Chem. Eng. J..

[36] Yang L., Yuan J., Wang G., Cao Q., Zhang C., Li M., Shao J., Xu Y., Li H., Lu J. (2023). Construction of Tri-Functional HOFs Material for Efficient Selective Adsorption and Photodegradation of Bisphenol A and Hydrogen Production. Adv. Funct. Mater..

[37] Sarkar R., Kundu T.K. (2020). Hydrogen Bond Interactions of Hydrated Aluminum Nitrate with PVDF, PVDF-TrFE, and PVDF-HFP: A Density Functional Theory-Based Illustration. Int. J. Quantum. Chem..

[38] Fortunato M., Chandraiahgari C.R., De Bellis G., Ballirano P., Sarto F., Tamburrano A., Sarto M.S. (2018). Piezoelectric Effect and Electroactive Phase Nucleation in Self-Standing Films of Unpoled PVDF Nanocomposite Films. Nanomaterials.

[39] Yuennana J., Muensit N. (2019). Fabrication and Properties of Flexible CaCl_2_/P(VDF-HFP) Composite Films. J. Phys. Conf. Ser..

[40] Sukwisute P., Yuennan J., Muensit N. (2018). Effects of the Electric Field and AlCl_3_·6H_2_O Salt on the Crystal, Morphology and Dielectric Properties of P(VDF-HFP) Fibres. J. Phys. Conf. Ser..

[41] Xue W., Lv C., Jing Y., Chen F., Fu Q. (2017). Fabrication of Electrospun PVDF Nanofibers with Higher Content of Polar β Phase and Smaller Diameter by Adding a Small Amount of Dioctadecyl Dimethyl Ammonium Chloride. Chin. J. Polym. Sci..

[42] Eleshmawi I.S. (2008). Effect of LiCl Filler on the Structure and Morphology of PVDF Films. J. Elastomers Plast..

[43] Tawansi A., Oraby A.H., Abdelrazek E.M., Ayad M.I., Abdelaziz M. (1998). Effect of Local Structure of MnCl_2_-Filled PVDF Films on Their Optical, Electrical, Electron Spin Resonance, and Magnetic Properties. J. Appl. Polym. Sci..

[44] Tawansi A., Ayad M.I., Abdel-Razek E.M. (1999). Effect of Valence Electron Spin Polarization on the Physical Properties of CuCl_2_-Filled Poly(Vinylidene Fluoride) as a Microwave Modulator. J. Appl. Polym. Sci..

[45] Tawansi A., Oraby A.H., Badr S.I., Elashmawi I.S. (2004). Physical Properties and β-Phase Increment of AgNO_3_-Filled Poly(Vinylidene Fluoride) Films. Polym. Int..

[46] Hakeem N.A., Abdelkader H.I., El-sheshtawi N.A., Eleshmawi I.S. (2006). Spectroscopic, Thermal, and Electrical Investigations of PVDF Films Filled with BiCl_3_. J. Appl. Polym. Sci..

[47] Elashmawi I.S. (2007). Effect of NaCl filler on ferroelectric phase and polaron configurations of PVDF films. Cryst. Res. Technol. J. Exp. Ind. Crystallogr..

[48] Chen C., Bai Z., Cao Y., Dong M., Jiang K., Zhou Y., Tao Y., Gu S., Xu J., Yin X. (2020). Enhanced Piezoelectric Performance of BiCl_3_/PVDF Nanofibers-Based Nanogenerators. Compos. Sci. Technol..

[49] Dhakras D., Borkar V., Ogale S., Jog J. (2012). Enhanced Piezoresponse of Electrospun PVDF Mats with a Touch of Nickel Chloride Hexahydrate Salt. Nanoscale.

[50] Ghosh S.K., Biswas A., Sen S., Das C., Henkel K., Schmeisser D., Mandal D. (2016). Yb^3+^ Assisted Self-Polarized PVDF Based Ferroelectretic Nanogenerator: A Facile Strategy of Highly Efficient Mechanical Energy Harvester Fabrication. Nano Energy.

[51] Orudzhev F., Alikhanov N., Amirov A., Rabadanova A., Selimov D., Shuaibov A., Gulakhmedov R., Abdurakhmanov M., Magomedova A., Ramazanov S. (2023). Porous Hybrid PVDF/BiFeO_3_ Smart Composite with Magnetic, Piezophotocatalytic, and Light-Emission Properties. Catalysts.

[52] Orudzhev F., Ramazanov S., Sobola D., Kaspar P., Trčka T., Částková K., Kastyl J., Zvereva I., Wang C., Selimov D. (2021). Ultrasound and Water Flow Driven Piezophototronic Effect in Self-Polarized Flexible α-Fe_2_O_3_ Containing PVDF Nanofibers Film for Enhanced Catalytic Oxidation. Nano Energy.

[53] Singh P., Borkar H., Singh B.P., Singh V.N., Kumar A. (2014). Ferroelectric Polymer-Ceramic Composite Thick Films for Energy Storage Applications. AIP Adv..

[54] Lotfian S., Giraudmaillet C., Yoosefinejad A., Thakur V.K., Nezhad H.Y. (2018). Electrospun Piezoelectric Polymer Nanofiber Layers for Enabling in Situ Measurement in High-Performance Composite Laminates. ACS Omega.

[55] Hartono A., Satira S., Djamal M., Ramli R., Bahar H., Sanjaya E., Hartono A., Satira S., Djamal M., Ramli R. (2013). Effect of Mechanical Treatment Temperature on Electrical Properties and Crystallite Size of PVDF Film. Adv. Mater. Phys. Chem..

[56] Constantino C.J.L., Job A.E., Simões R.D., Giacometti J.A., Zucolotto V., Oliveira O.N., Gozzi G., Chinaglia D.L. (2005). Phase Transition in Poly(Vinylidene Fluoride) Investigated with Micro-Raman Spectroscopy. Appl. Spectrosc..

[57] Barnakov Y.A., Paul O., Joaquim A., Falconer A., Barnakov V.Y., Dikin D., Petranovskii V.P., Zavalin A., Ueda A., Williams F. (2018). Light Intensity-Induced Phase Transitions in Graphene Oxide Doped Polyvinylidene Fluoride. Opt. Mater. Express.

[58] Chen S., Yao K., Tay F.E.H., Liow C.L. (2007). Ferroelectric Poly(Vinylidene Fluoride) Thin Films on Si Substrate with the Β Phase Promoted by Hydrated Magnesium Nitrate. J. Appl. Phys..

[59] Hare D.E., Sorensen C.M. (1992). Interoscillator Coupling Effects on the OH Stretching Band of Liquid Water. J. Chem. Phys..

[60] Carey D.M., Korenowski G.M. (1998). Measurement of the Raman Spectrum of Liquid Water. J. Chem. Phys..

[61] Brooksby P.A., Ronald Fawcett W. (2000). Infrared (ATR) Study of Hydrogen Bonding in Solutions Containing Water and Ethylene Carbonate. J. Phys. Chem. A.

[62] Mohammadi Ghaleni M., Al Balushi A., Kaviani S., Tavakoli E., Bavarian M., Nejati S. (2018). Fabrication of Janus Membranes for Desalination of Oil-Contaminated Saline Water. ACS Appl. Mater. Interfaces.

[63] Mi C., Ren Z., Li H., Yan S., Sun X. (2019). Synergistic Effect of Hydrogen Bonds and Diffusion on the β-Crystallization of Poly(Vinylidene Fluoride) on Poly(Methyl Methacrylate) Interface. Ind. Eng. Chem. Res..

[64] Jana S., Garain S., Sen S., Mandal D. (2015). The Influence of Hydrogen Bonding on the Dielectric Constant and the Piezoelectric Energy Harvesting Performance of Hydrated Metal Salt Mediated PVDF Films. Phys. Chem. Chem. Phys..

[65] Wang H., Jin S., Zhang X., Xie Y. (2020). Excitonic Effects in Polymeric Photocatalysts. Angew. Chem. Int. Ed..

[66] Bakulin A.A., Rao A., Pavelyev V.G., Van Loosdrecht P.H.M., Pshenichnikov M.S., Niedzialek D., Cornil J., Beljonne D., Friend R.H. (2012). The Role of Driving Energy and Delocalized States for Charge Separation in Organic Semiconductors. Science.

[67] Balzer D., Kassal I. (2022). Even a Little Delocalization Produces Large Kinetic Enhancements of Charge-Separation Efficiency in Organic Photovoltaics. Sci. Adv..

[68] Ononye A.I., McIntosh A.R., Bolton J.R. (1986). Mechanism of the Photochemistry of P-Benzoquinone in Aqueous Solutions. 1. Spin Trapping and Flash Photolysis Electron Paramagnetic Resonance Studies. J. Phys. Chem..

